# Moving beyond the hospital: in-depth characterization of daily-life mobility in patients with atypical Parkinsonian disorders

**DOI:** 10.1038/s41531-025-01242-2

**Published:** 2026-01-12

**Authors:** Victoria Sidoroff, Hamid Moradi, Gaëlle Prigent, Frank Jagusch, Isabelle Teckenburg, Marzieh Asalian, Nina Hergenroeder-Lenzner, Marijus Giraitis, Eva Tabea Schoenfeldt-Reichmann, Jean-Pierre Ndayisaba, Georg Goebel, Klaus Seppi, Anisoara Ionescu, Florian Krismer, David Benninger, Juergen Winkler, Bjoern M. Eskofier, Jochen Klucken, Kamiar Aminian, Gregor Wenning, Stefano Sapienza, Heiko Gassner, Cecilia Raccagni, MobilityAPP Study Group

**Affiliations:** 1 Department of Neurology, Medical University of Innsbruck, Innsbruck, Austria; 2 Machine Learning and Data Analytics Lab, Friedrich-Alexander-University Erlangen-Nürnberg, Erlangen, Germany; 3 Laboratory of Movement Analysis and Measurement, École Polytechnique Fédérale de Lausanne, Lausanne, Switzerland; 4 Department of Molecular Neurology, University Hospital Erlangen, Erlangen, Germany; 5 University of Luxembourg, Esch-sur-Alzette, Luxembourg; 6 Neurology, Centre Hospitalier Universitaire Vaudois, Lausanne, Switzerland; 7 Université de Lausanne, Lausanne, Switzerland; 8 Neurology, Reha Rheinfelden, Rheinfelden, Switzerland; 9 Center for Rare Diseases Erlangen (ZSEER), University Hospital Erlangen, Erlangen, Germany; 10 Institute of AI for Health, Helmholtz Zentrum München-German Research Center for Environmental Health, Neuherberg, Germany; 11 Centre Hospitalier de Luxembourg, Luxembourg City, Luxembourg; 12 Fraunhofer IIS, Fraunhofer Institute for Integrated Circuits IIS, Erlangen, Germany; 13 Department of Neurology, Provincial Hospital of Bolzano, Bolzano, Italy; 14 Paracelsus Private Medical University, Salzburg, Austria

**Keywords:** Neurology, Neurological disorders

## Abstract

This study evaluates mobility in patients with multiple system atrophy (MSA), progressive supranuclear palsy (PSP), and Parkinson’s disease (PD) by integrating clinical assessments, instrumented gait analysis (IGA) in the hospital, and 1 week of physical activity monitoring (PAM) at home, using wearable sensors. Clinical scores provide a broad measure of disease severity but lack precision in quantifying gait impairments. IGA offers objective gait metrics under standardized conditions, identifying deficits in stride dynamics and postural control. However, these controlled assessments do not reflect real-world mobility. PAM addresses this gap by continuously tracking movement patterns and physical activity during daily-life, offering insights into how patients walk beyond clinical settings. The combination of IGA and PAM provides a more comprehensive understanding of mobility limitations, particularly in MSA and PSP, where gait and balance impairments differ from PD. This dual approach enhances patient assessment, supports personalized disease management, and improves clinical decision-making. Trial registration: ClinicalTrials.gov, NCT04608604, date of registration: 19/10/2020, first patient enrollment: 01/02/2021.

## Introduction

Axial impairments, such as postural instability and gait difficulties (PIGD), are key features of parkinsonian syndromes. These motor symptoms are more prominent in atypical parkinsonian disorders (APD), such as multiple system atrophy (MSA) and progressive supranuclear palsy (PSP), leading to falls, injuries, and reduced quality of life. While gait in patients with idiopathic Parkinson’s disease (PD) is characterized by a narrow base and short steps, patients with MSA usually show a broader base due to pronounced instability in the mediolateral plane^1,2^. Gait in patients with PSP is also mainly broad-based but consists of quick and reckless movements that are often poorly controlled and result in impaired balance, causing mostly backward falls^2–4^. In contrast to PD patients, that mainly develop gait difficulties years after symptom onset, APDs develop axial symptoms in a very early disease stage and progress in a much faster way. Our knowledge about detailed insight about gait characteristics in APD is lacking due to the rare disease status of MSA and PSP making it difficult to characterize and—in a next step—measure improvement after therapies.

PIGD symptoms are typically assessed individually in clinical practice using validated rating scales. However, these scales are rater-dependent and require a trained physician for the evaluation. For this reason, they are largely limited to hospital settings, which represents only a snapshot of the patient’s clinical state in a controlled environment. Consequently, they may not adequately capture the full spectrum of gait and mobility difficulties these populations face. For this reason, the importance of reliable, objective, continuous, and potentially real-time outcome measures has grown rapidly^5^.

In the last decade, inertial measurement units (IMUs) equipped with three-dimensional accelerometers, gyroscopes, and magnetometers have provided evidence to objectively measure clinically relevant gait characteristics and daily activities at various levels in different neurological diseases^6–13^. Although investigated only in small cohorts, few studies have shown that MSA^14–16^ and PSP^15,17,18^ patients exhibit different gait patterns during supervised short walking tests in the clinic, with mainly shorter stride length and higher gait variability. Additionally, the current knowledge of daily-life mobility in these populations remains primarily based on patient- and caregiver-reported information and, to the best of our knowledge, with no evidence available on home-based sensor-supported analyses of PIGD symptoms focusing on patients with MSA and PSP. Due to the fluctuating and often unpredictable nature of motor symptoms in parkinsonism, the objective investigation of how gait difficulties influence everyday mobility in APD and their link with standard clinical ratings is paramount since it properly reflects the real-life condition of these patients, with the opportunity to gain a better understanding of the patients’ mobility.

Given these premises, this manuscript aims to assess mobility in a well-characterized cohort of patients with MSA, PSP, and PD by investigating via a rigorous statistical analysis:

1. differences between groups in sensor-derived walking parameters extracted during an instrumented gait analysis (IGA) performed in the clinic using foot-worn IMUs,
2. differences between groups in daily-life mobility metrics assessed in a week of physical activity monitoring (PAM) using the same devices, and
3. correlations for each group between both measurements and clinical reference -scores collected in the hospital to interpret their usage and value.

For answering those questions, our cohort underwent a full clinical and cognitive examination, followed by IGA and PAM analysis. Further details are described in the “Methods” section. We hypothesize that PAM data provides added value to comprehensively characterize mobility patterns in PD, MSA, and PSP.

## Results

### Study population

A total of 106 patients (33 MSA, 26 PSP, and 47 PD) were screened for this analysis. One MSA patient dropped out during the home recording phase. After applying the minimum PAM recording criteria based on recommendations from the literature^19,20^, at least 3 days with 8 h of daily wearing-time, 18 participants were excluded due to insufficient recording. Additionally, three other participants were excluded due to technical issues in the IGA recordings. Finally, 84 patients (23 MSA, 20 PSP, and 41 PD) were included in this analysis. The detailed flowchart and process of data collection is presented in Fig. 4C.

All the significant levels of all statistical tests and correlations are defined at *p* values below the threshold (*α* < 0.05) after being adjusted for the false discovery rate (FDR) according to Benjamini–Hochberg’s correction. The correlations with *ρ* ≤ 0.25 were classified as weak, 0.25–0.50 as moderate, 0.50−0.75 as strong, and ≥0.75 as very strong.

### Demographics and clinical characterization

All patients with MSA and PSP who met the inclusion criteria and consented to participate were enrolled in the study. Patients with PD were age- and sex-matched to the APD cohort. Patients with MSA and PSP had a shorter disease duration and higher motor and balance impairment compared to PD. Table 1 presents the cohort’s demographic data and clinical characteristics.

**Table 1:** Demographic and clinical data

Parameter	MSA (*n* = 23)	PSP (*n* = 20)	PD (*n* = 41)	*p* value^#^	Effect size	*p* value MSA-PSP	*p* value MSA-PD	*p* value PSP-PD
Age	64.7 (±7.2)	68.7 (±6.4)	68.9 (±8.5)	0.13	0.05	NA	NA	NA
Sex (% female)	52.2	30.0	43.9	0.24	0.01	NA	NA	NA
Disease Duration (y)	4 (3)	3 (3)	6 (9)	**0.001**	0.16	–	**	***
MoCA	27.0 (3)	23.0 (4)	27.0 (4)	**0.006**	0.10	*	–	***
FAB	16.0 (4)	13.5 (3)	17.0 (2)	**<0.001**	0.18	*	*	***
PDQ-8	11.0 (10)	10.5 (11)	3.0 (6)	**<0.001**	0.22	–	***	**
OH (%)	73.9	25.0	14.6	**<0.001**	0.23	**	***	–
Hoehn & Yahr	3.0 (0.5)	3.0 (2)	2.0 (0)	**<0.001**	0.36	–	***	***
MDS-UPDRS I	13 (9)	9 (6)	8 (10)	**0.001**	0.13	**	***	–
MDS-UPDRS II	22 (15)	16.5 (11)	9 (9)	**<0.001**	0.30	–	***	***
MDS-UPDRS III	39 (19)	40.5 (22)	18 (14)	**<0.001**	0.35	–	***	***
MDS-UPDRS Total	77 (35)	55.5 (31.5)	37 (24)	**<0.001**	0.37	–	***	***
PIGD Subscore	6.0 (±2.5)	6.7 (±3.5)	2.2 (±1.9)	**<0.001**	0.41	–	***	***
Fallers (%)	39.1	60.0	7.3	**0.002**	0.14	*	–	***
BBS	47 (10)	46 (11)	55 (3)	**<0.001**	0.36	–	***	***
TUG	13.4 (4)	15.7 (7)	9.3 (4)	**<0.001**	0.25	–	***	***
IPAQ Walking	495 (1225.1)	313.5 (878.6)	1386 (1889.2)	**0.005**	0.10	–	**	**
IPAQ Total	1367.3 (1776)	1077 (2742.8)	3463.5 (4531.9)	**<0.001**	0.20	–	***	***

Based on the distributions, values are reported either as the mean (±standard deviation) or as the median (interquartile range).

*MoCA* Montreal Cognitive Assessment, *FAB* frontal assessment battery, *PDQ-8* Parkinson’s disease questionnaire, *OH* orthostatic hypotension, *MDS-UPDRS* Movement disorder society Unified Parkinson’s disease rating scale, *PIGD* postural instability and gait difficulty, *BBS* Berg balance scale, *TUG* timed up and go test, *IPAQ* International Physical Activity Questionnaire.

^#^*p* values adjusted for the false discovery rate (FDR) according to Benjamini–Hochberg’s correction. *p < 0.05; **p < 0.01; ***p < 0.001.

Numbers in bold indicate significance.

### IGA results

Table 2 shows the differences among the three groups of MSA, PSP, and PD in the sensor-derived gait parameters collected during IGA. The APD groups presented a significantly lower self-selected gait velocity in the two-times-ten-meter walk test compared to the PD group. They also showed a shorter stride length and, longer stride time. Patients with PSP showed a significantly higher stance percentage, whereas the maximum sensor lift was significantly lower in patients with MSA.

**Table 2:** Gait parameters derived from IGA - 2x10m test

Parameter	MSA (*n* = 23)	PSP (*n* = 20)	PD (*n* = 41)	*p* value^#^	Effect size	*p* value MSA-PSP	*p* value MSA-PD	*p* value PSP-PD
Mean								
Gait Velocity (m/s)	0.90 (0.27)	0.91 (0.23)	1.24 (0.27)	**<0.001**	0.34	–	***	***
Stride length (m)	0.99 (0.31)	1.10 (0.22)	1.31 (0.25)	**<0.001**	0.26	–	***	***
Stride time (s)	1.15 (0.15)	1.28 (0.22)	1.08 (0.08)	**0.002**	0.13	–	**	***
Stance percentage	63.77 (2.41)	65.42 (2.89)	63.72 (2.07)	**0.013**	0.08	*	–	**
Max. sensor lift (m)	0.05 (0.03)	0.06 (0.02)	0.07 (0.02)	**0.001**	0.17	*	***	–
Coefficient of variation								
Gait Velocity	0.09 (0.03)	0.11 (0.07)	0.08 (0.02)	**0.002**	0.14	–	**	***
Stride length	0.07 (0.02)	0.07 (0.05)	0.05 (0.02)	**<0.001**	0.18	–	***	**
Stride time	0.05 (0.02)	0.07 (0.03)	0.04 (0.03)	**0.001**	0.16	*	*	***
Stance percentage	0.04 (0.02)	0.04 (0.02)	0.02 (0.02)	**0.010**	0.09	–	**	**
Max. sensor lift	0.17 (0.13)	0.14 (0.06)	0.13 (0.04)	0.080	0.03	NA	NA	NA
Asymmetry (%)								
Gait Velocity	3.91 (1.64)	3.85 (1.98)	2.91 (0.77)	**0.001**	0.16	–	***	***
Stride length	3.91 (2.14)	3.30 (1.98)	2.55 (1.16)	**0.001**	0.14	–	***	**
Stride time	1.64 (1.86)	2.15 (1.48)	1.63 (0.89)	**0.008**	0.10	*	–	***
Stance percentage	3.43 (1.69)	2.92 (2.09)	2.15 (1.64)	**0.009**	0.09	–	**	**
Max. sensor lift	19.76 (12.49)	15.37 (6.00)	13.17 (10.35)	**0.048**	0.05	–	**	–

Values are reported as median and interquartile range.

^#^*p* values adjusted for the false discovery rate (FDR) according to Benjamini–Hochberg’s correction. *p < 0.05; **p < 0.01; ***p < 0.001.

Numbers in bold indicate significance.

**Fig. 1:**
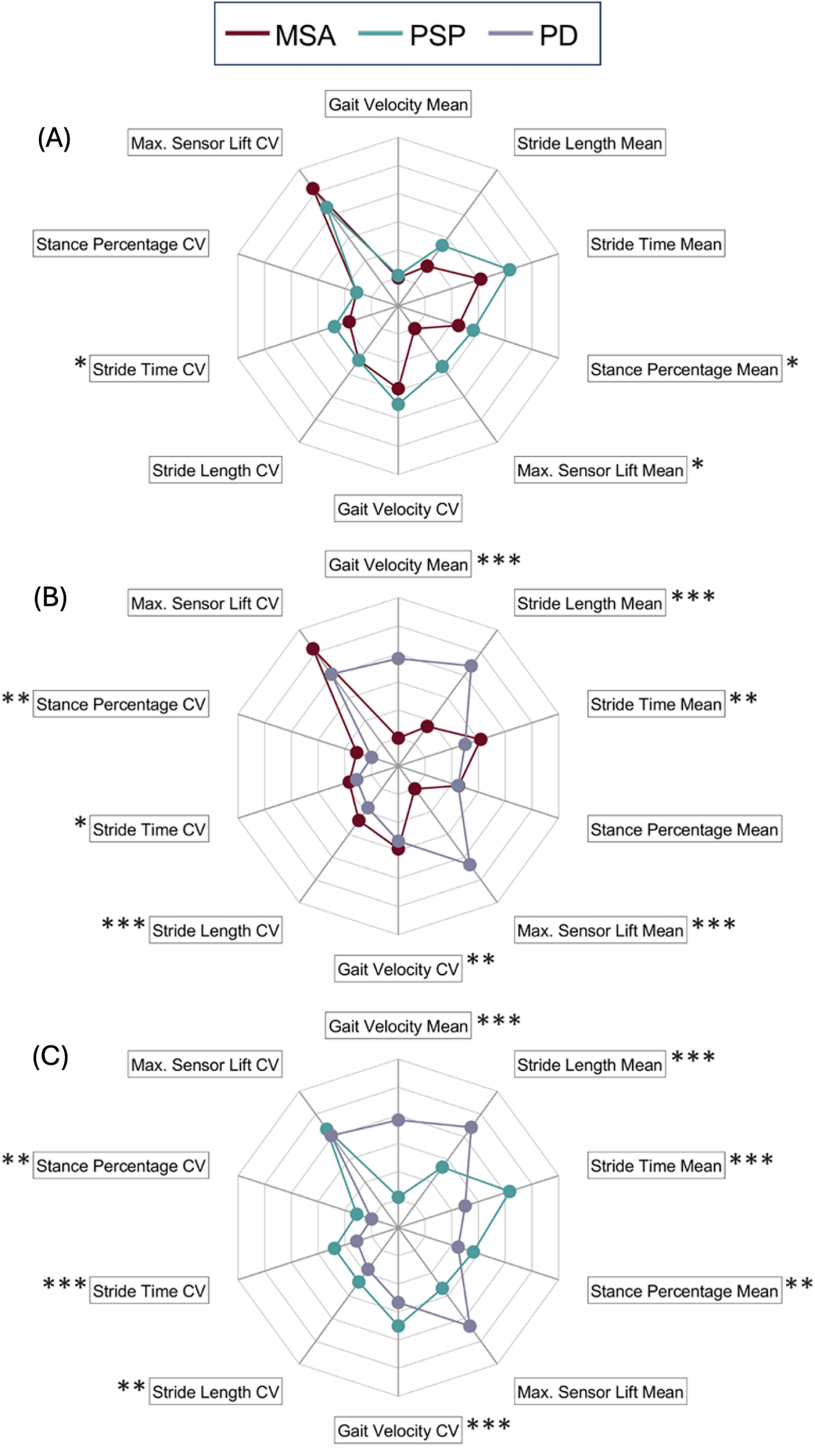
Comparison of sensor-derived gait parameters in the clinic. Group-wise comparisons of IGA (instrumented gait analysis at the clinic) between the three groups. **A** compares MSA and PSP, **B** compares MSA and PD and **C** compares PSP and PD patients. CV coefficient of variation. *p* values adjusted for the false discovery rate (FDR) according to Benjamini–Hochberg’s correction. *p < 0.05; **p < 0.01; ***p < 0.001. All the spider plots share the same axis. For readability purposes the unit of measurements are shown in Fig. 2 of the Supplementary Materials.

Patients with APD exhibited overall a higher variability compared to patients with PD, except for maximum sensor lift. Among the variability features, only the CV for stride time was significantly different when comparing the PSP group with the MSA group. Group-wise comparisons of mean and CV parameters between the three groups are visualized in Fig. 1 (the axis unit of measurements are shown in Supplementary Fig. 2 for readability purposes).

Finally, patients with APD showed higher asymmetry values across all parameters, with stride time asymmetry significantly higher in the PSP compared to the MSA group.

### PAM results

**Fig. 2:**
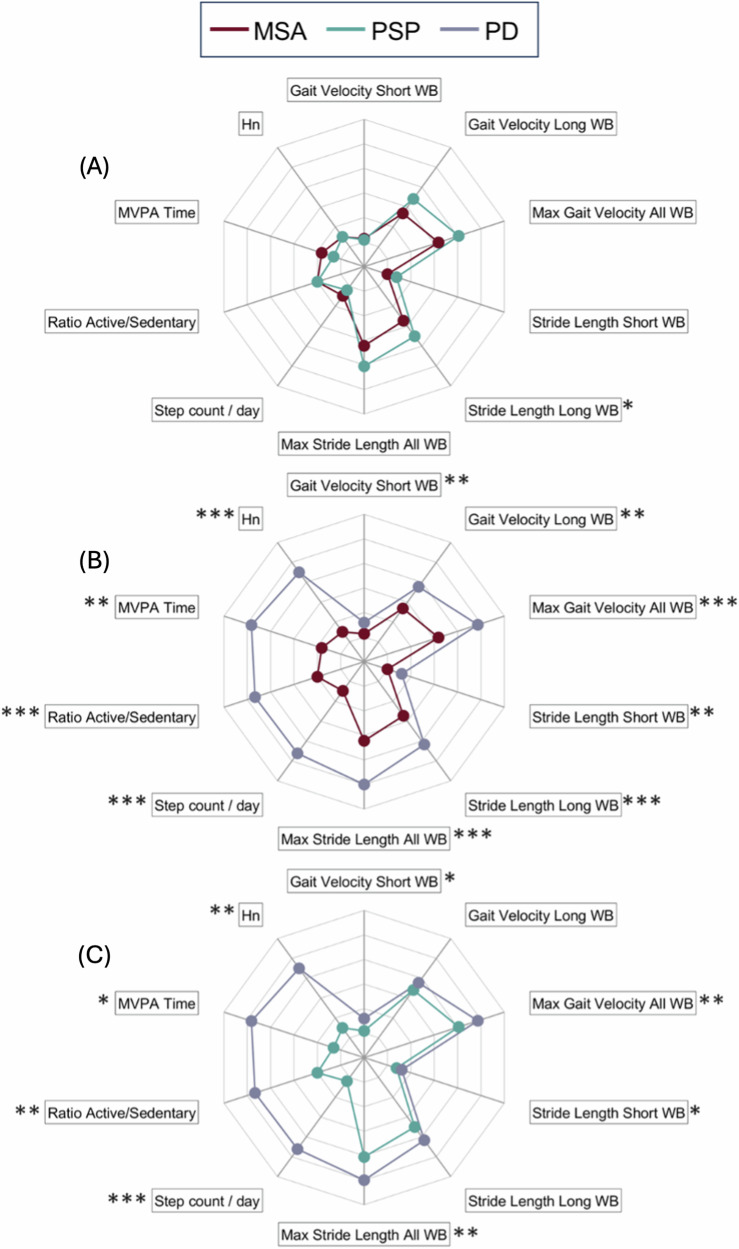
Comparison of sensor-derived gait parameters in daily-life. Group-wise comparisons of PAM (physical activity monitoring at home) between the three groups. **A** compares MSA and PSP, **B** compares MSA and PD and **C** compares PSP and PS patients. MVP moderate-to-vigorous physical activity, Hn information entropy. *p* values adjusted for the false discovery rate (FDR) according to Benjamini–Hochberg’s correction. * <0.05; **p < 0.01; ***p < 0.001. All the spider plots share the same axis. For readability purposes the unit of measurements are shown in Fig. 3 of the Supplementary Materials.

The extracted parameters from PAM recordings are presented in Table 3 and group-wise comparisons are visualized in Fig. 2 (the axis unit of measurements are shown in Supplementary Fig. 3 for readability purposes). In total, 496 valid days were analyzed, with an average of 5.5 ± 1.4 days per patient, similar across the three groups (PD: 5.8 ± 1.4; MSA: 5.5 ± 1.4; PSP: 5.0 ± 1.2, *p* = 0.16).

**Table 3:** PAM parameters recorded during daily living

Parameter	MSA (*n* = 23)	PSP (*n* = 20)	PD (*n* = 41)	*p* value^#^	Effect size	P MSA-PSP	P MSA-PD	P PSP-PD
Short WB (10–30 s)								
Gait velocity (m/s)	0.43 (0.13)	0.42 (0.15)	0.53 (0.17)	**0.020**	0.10	–	**	*
Stride length (m)	0.60 (0.17)	0.67 (0.17)	0.71 (0.22)	**0.027**	0.07	–	**	*
Cadence (step/min)	93.78 (12.56)	89.02 (17.75)	95.65 (11.82)	0.265	0.01	NA	NA	NA
WB /day	108.50 (46.75)	92.50 (69.88)	137.00 (61.00)	**0.027**	0.07	–	*	**
Medium WB (31–60 s)								
Gait velocity (m/s)	0.50 (0.15)	0.54 (0.20)	0.64 (0.22)	**0.020**	0.09	–	**	*
Stride length (m)	0.69 (0.13)	0.72 (0.23)	0.82 (0.23)	0.065	0.08	NA	NA	NA
Cadence (step/min)	93.22 (11.23)	86.96 (19.11)	91.96 (11.76)	0.471	0.00	NA	NA	NA
WB / day	12.00 (7.50)	11.25 (17.00)	14.50 (8.50)	0.294	0.00	NA	NA	NA
Long WB (>60 s)								
Gait velocity (m/s)^a^	0.77 (0.24)	0.93 (0.41)	1.01 (0.22)	**0.020**	0.12	–	**	–
Stride length (m)^a^	0.91 (0.16)	1.05 (0.34)	1.17 (0.22)	**0.008**	0.14	*	***	–
Cadence (step/min)^a^	98.23 (8.31)	98.79 (22.95)	104.62 (10.32)	0.243	0.01	NA	NA	NA
WB/day	3.50 (6.50)	6.00 (4.25)	7.00 (5.00)	**0.027**	0.07	–	**	–
Max N of WB/day	9.00 (9.50)	13.00 (8.25)	14.00 (10.00)	**0.027**	0.07	–	**	*
Duration of WB (s)	92.19 (35.61)	99.92 (73.97)	110.25 (49.59)	0.093	0.04	NA	NA	NA
95th percentile All WB								
Gait velocity (m/s)	0.88 (0.33)	1.07 (0.34)	1.25 (0.27)	**0.001**	0.19	–	***	**
Stride length (m)	1.00 (0.37)	1.15 (0.36)	1.32 (0.19)	**0.001**	0.18	–	***	**
Macro and complexity parameters (median)								
Step count/day	6471 (3221)	6189 (3762)	9614 (3835)	**0.001**	0.20	–	***	***
Ratio active/sedentary	0.13 (0.06)	0.13 (0.11)	0.21 (0.12)	**0.008**	0.12	–	***	**
MVPA time (min/day)	24.88 (20.67)	21.85 (42.86)	42.92 (40.80)	**0.018**	0.10	–	**	*
Information Entropy	0.21 (0.08)	0.21 (0.10)	0.27 (0.10)	**0.008**	0.12	–	***	**

Each data point represents the median value of the feature for each patient, except the “Max N of WB/day” which is the maximum number of long walking bouts per day. Values are reported as the median with the interquartile range.

*WB* walking bout, *WB/day* number of walking bouts per day, *MVPA* moderate-to-vigorous physical activity.

^#^*p* values adjusted for the false discovery rate (FDR) according to Benjamini–Hochberg’s correction. *p < 0.05; **p < 0.01; ***p < 0.001.

^a^Due to the defined minimum of 10 long WB for each subject, those who did not have enough data were excluded from the statistical analysis. MSA: *n* = 18, PSP: *n* = 18, PD: *n* = 39.

Numbers in bold indicate significance.

Compared to the PD group, the APD groups had a significantly smaller median number of steps and short WBs, along with a lower maximum number of long WBs. This contributed to the significantly lower active-to-sedentary ratio in patients with MSA and PSP. Furthermore, the moderate-to-vigorous physical activity (MVPA) period—i.e., moments of intense locomotion with a cadence higher than 90 steps per minute—was also significantly shorter in the APD groups.

In terms of micro parameters, patients with PD walked significantly faster and had longer stride lengths during short WBs compared to those with MSA and PSP. As the duration of WBs considered increases, mixed effects are observed. For medium WBs, higher velocity but similar stride length was measured between the APD and PD groups. In contrast, patients with MSA exhibited a significantly shorter stride length compared to both PSP and PD during long WBs. The most pronounced differences were observed in the 95th percentile gait velocity and stride length, where a clear distinction was evident between the APD and PD groups. Additionally, the complexity of daily-life mobility patterns was reduced in the APD groups, as highlighted by the smaller values of Information Entropy (Hn) in patients with MSA and PSP compared to those with PD.

### Correlation analysis

**Fig. 3:**
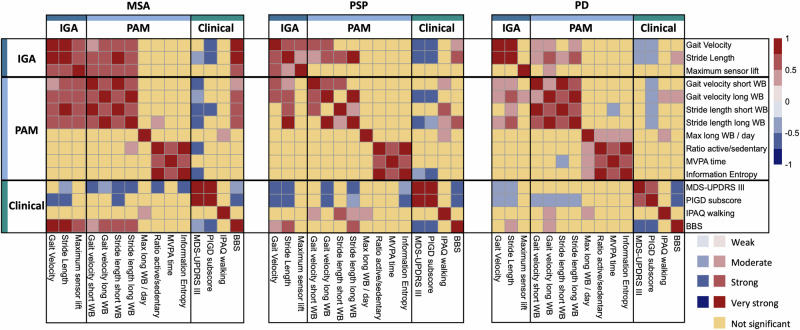
Correlation heatmap across the domains. Group-wise correlation heatmap between IGA parameters, PAM parameters, and clinical measurements. Correlation coefficients (*ρ*) are categorized as weak (*ρ* ≤ 0.25), moderate (0.25 < *ρ* ≤ 0.50), strong (0.50 < *ρ* ≤ 0.75), and very strong (*ρ* > 0.75). IGA instrumented gait analysis in the clinic, PAM physical active monitoring at home, MDS-UPDRS movement disorder society Unified Parkinson’s disease rating scale, PIGD postural instability and gait difficulty, IPAQ International physical activity questionnaire, BBS Berg balance scale, MVPA moderate to vigorous physical activity.

The correlations were performed among (1) the three IGA parameters that showed the most significant statistical differences (effect sizes) and clinically meaningful differences between the three groups—average *gait speed*, and average *stride length* reflecting walking performance and average *maximum sensor lift* which represents an important indicator of risk of falls—, (2) four clinical scores related to motor deficits—MDS-UPDRS III for general motor impairment, PIGD for axial impairment, BBS for balance, and IPAQ walking for PROM of daily activity—and (3) PAM features, representing both micro-parameters and macro-parameters as a broader measure of physical activity at home. The results of these correlations are presented in Fig. 3.

The gait parameters obtained from IGA showed comparable patterns across groups, with the mean gait velocity and stride length exhibiting the largest coefficients. The gait velocity had moderate to strong correlations with the MDS-UPDRS III (${\rho }_{{MSA}}$ = −0.40, ${\rho }_{{PD}}$ = −0.48, ${\rho }_{{PSP}}$ = −0.67), and even higher with the PIGD sub-scores (${\rho }_{{MSA}}$ = −0.62, ${\rho }_{{PD}}$ = −0.50, ${\rho }_{{PSP}}$ = −0.71). Strong to very strong positive correlations were found between the stride length of IGA and the BBS ratings (${\rho }_{{MSA}}\,$= 0.80, ${\rho }_{{PD}}$ = 0.39, ${\rho }_{{PSP}}$ = 0.57). Among the three groups, patients with MSA presented the highest correlation coefficients across all the clinical scales.

In terms of macro parameters, the maximum number of long walking bouts (WBs) per day showed a moderate to strong correlation with the IPAQ walking score across all groups. Also, the median number of long WBs per day in the patients with PSP and PD showed a strong and moderate correlation with IPAQ respectively. In patients with PSP, strong correlations between MDS-UPDRS III and PIGD scores, and Hn metrics were observed. While in patients with MSA active vs. sedentary ratio and Hn strongly correlated to MDS-UPDRS III.

For the micro parameters, the 95^th^ percentile gait velocity and stride length of all WBs were the two features correlating significantly across all three groups when associated with the PIGD score. Mixed behaviors were observed with MDS-UPDRS III. Patients with MSA presented significant coefficients in gait velocity and stride length for all the WB types, PSP patients only for long WB, and PD for none of them (Table 3 in Supplementary Materials).

While gait velocity and stride length in APD presented a strong or very strong correlation between lab measurements and long WBs at home (${\rho }_{{MSA}}$ = 0.55, ${\rho }_{{PSP}}$ = 0.75), PD only showed a moderate correlation (${\rho }_{{PD}}=0.44$). It is worth mentioning that the correlation between the gait velocity at lab is higher with long WB compared to short WB in all groups. Additionally, there are no associations between the IGA parameters and macro parameters obtained from PAM.

## Discussion

Given the heterogeneous and multifaceted nature of parkinsonian syndromes, a comprehensive understanding of these diseases is necessary. To achieve the entire picture of the disease, metric mobility data captured in standardized gait assessments in the hospitals as well as continuous recordings from daily-life complement the clinical presentation as well as PROMs. The complex interplay between these three domains may provide valuable insights to understand parkinsonian diseases and their therapy response. This manuscript addresses these three pillars, and two are the main contributions of this work. First, the quantification of daily-life mobility in patients with APD, which allows to monitor gait beyond the hospital wall. This opens the possibility to measure crucial aspects of daily living, that, however, would be impossible to properly interpret and contextualize without the clinical and IGA information collected in the hospital as essential reference.

The second contribution of the manuscript lies in the size of the APD cohort itself. In contrast to previous studies that, due to smaller sample sizes, pooled patients with APD together^14,15,17,18,21,22^, this study evaluates patients with MSA and PSP separately. This may allow to address the disease-specific footprints, which may otherwise escape the naked clinical eye.

The importance of this separation is immediately evident already from the first level of characterization: the clinical assessment. While shorter disease duration and higher MDS-UPDRS III scores in patients with APD are well-known and reflect the rapid progression despite L-Dopa intake, other disease-specific factors differentiate MSA from PSP, highlighting that the impairment extends beyond the motor domain. Higher impairment in cognition as presented in PSP, mainly described as dysexecutive function, together with impulsivity and lack of awareness of instability lead to higher fall frequency and less autoregulation of walking, for example walking slower to enhance security^23,24^. Whereas on the other hand, MSA patients with 39.1% of fallers are potentially mainly influenced by autonomic dysfunction with 78% of MSA fallers were simultaneously diagnosed with OH. This highlights the substantial role of autonomic dysfunction, particularly OH, which may affect gait performance in patients with PD and MSA, as suggested by previous work^25^.

If clinical scales and PROMs lay the foundation of gait characterization, which visually or subjectively assess motor impairment, IGA allows to objectively identify walking patterns during standardized tasks through quantifiable spatiotemporal parameters.

The statistical analysis of the sensor-derived features collected in the clinic showed a clear difference between patients with APD and PD in most spatiotemporal gait parameters. Patients with APD exhibited significantly lower gait velocity, shorter stride length, and longer stride time^15^. These features were also associated with higher values of inter-stride variability and asymmetry, indicating a greater gait disturbance in patients with APD. The results align with previous smaller studies^16,26^, confirming the differences captured by clinical scores during the visits.

Separate investigations of patients with MSA and PSP provide further insight into the distinct gait patterns of these two groups, which in our cohort had similar MDS-UPDRS III and PIGD scores, showing no significant differences.

Disease-specific differences in IGA parameters included a larger stance phase percentage, stride time variability, and asymmetry for patients with PSP compared to those with MSA. These findings confirm previous observations that prolonged stance is a typical gait characteristic seen early in patients with PSP^21^. Additionally, gait variability is a primary indicator of effective postural control and reduced gait stability^27^.

Furthermore, the maximum sensor lift is another signature gait parameter detected by the sensor recordings. In normative gait, this feature serves as a proxy of foot elevation. The gait behavior observed in patients with MSA is characterized by lower velocity and significantly smaller values of maximum sensor lift compared to those with PD and may be relevant to the digital observation of trip and fall risk. Nonetheless, this does not occur in participants with PSP, who tend to raise their feet higher. Although PSP patients have the highest percentage of falls compared to MSA and PD, this suggests that the falls occurring in PSP patients are not due to tripping over obstacles. If confirmed by future studies, the maximum sensor lift could serve as a distinctive parameter for distinguishing the ambulation patterns of these two APD populations.

The third level of gait characterization is the assessment of daily-life mobility that complements the detailed in-hospital investigation by adding details about unsupervised real-life mobility and may also catch information which eludes during clinical evaluation, due to the unpredictable and fluctuating nature of parkinsonian syndromes.

Ambulation in the real world is generally influenced by additional variability due to the complex interplay of environmental factors, including turns, curved paths, multitasking demands, and obstacles. In these conditions, stratifying by WB duration is crucial for better interpreting the results^11^. Short and medium WBs are predominantly performed indoors in a constrained environment. Conversely, longer WBs typically present fewer external perturbations, allowing for more steady-state velocity. Thus, they are more closely related to the individual’s standard gait patterns and what is observed in clinical settings^11^.

The statistical analysis of macro parameters facilitates the accurate quantification of daily-life mobility in patients with APD. Specifically, the step count per day, MVPA time, and active-to-sedentary ratio clearly highlight the more active daily-life of patients with PD compared to those with MSA and PSP. This concept is further underscored by the significantly lower information entropy in patients with APD, emphasizing that the differences are not solely dependent on walking volume and rhythms but also on the variety and diversity of daily activities. It is worth noting that the PD participants in the study, with a median number of daily steps slightly above 9.500 should be considered active according to literature standards^28^. Although the number of steps in this population might be influenced by the shuffling gait, we speculate that the participation in the MobilityAPP trial, due to its intense physical intervention, attracted individuals with PD who were moremobile than average. Future studies should verify if this applies also to our APD population and if the PAM metrics measured in this manuscript represent rather an upper limit than the standard behavior.

Micro parameters complement the physical activity information by providing a synergistic perspective through the characterization of walking patterns in daily-life. Patients with APD demonstrate differences in stride length and gait velocity but not in cadence. When considering the long WBs, comparable walking speeds were observed between patients with PSP and PD despite their differing clinical characteristics, also correlating with IGA and PAM metrics. To possibly explain this phenomenon, it is important to consider the results of the IGA tests. The gait velocity during IGA in patients with PSP is similar to that measured during long WBs, while patients with MSA and PD walk much slower in daily-life scenarios. This suggests that patients with PSP are less able or inclined to modulate their speed according to environmental conditions or to external cues. This finding aligns with the “careless walking” pattern^4^ known in this population, who is unaware of its increased fall risk due to the affection of the prefrontal cortex. This is relevant to further investigate e.g. fall risk patterns in daily-life using IMUs to have the chance to early detect subtle changes of gait patterns, prevent falls, and comorbidities using digital technologies.

Finally, in our cohort, patients with PD reached a higher maximum speed during daily-life compared to those with APD. Although this result should be interpreted in the context of the different impairment levels of both groups, this maximum performance metric is particularly important as it means the actual maximum speed patients can voluntarily achieve and therefore reflect performance. Previous studies on other diseases have shown that a limited ability to increase walking speed is a significant concern and can lead to activity avoidance^29^.

Since clinical scores, IGA, and PAM provide different conceptual perspectives on gait characterization, it is essential to investigate how information from these three domains is interconnected for a proper interpretation of the results.

When evaluating correlations between clinical assessments and IGA parameters, the mean gait velocity and stride length showed moderate to strong within-group correlation values with clinical motor scores, in line with previous findings^15^. The higher coefficient values, particularly in PIGD, observed in patients with APD underline that the axial component impacts this population more than PD^30^.

The results on the PAM micro-parameters showed that for patients with APD, the maximum speed, maximum stride length, and median stride length in long WBs are correlated with the MDS-UPDRS III and PIGD scores. These findings demonstrate that, in this group, maximum gait performance and long WBs in daily-life reflect the motor and gait difficulties assessed by clinicians in the hospital. Consequently, such PAM-derived parameters allow clinicians to assess mobility over longer periods outside the clinical setting. These objective measures may provide a cost-effective means of generating comprehensive dataset that can be used for in-depth analysis and therapy planning.

Among the PAM macro-parameters, only the median number of long WBs presented a significant correlation with the IPAQ walking self-reported score across all three groups. This suggests that the self-reported PA is mainly based on long durations of activities. In contrast, shorter and medium WBs, despite being more frequent, seem to be overlooked in self-reports. A reason for this might be that these questionnaires are not designed to separate between WB lengths as defined by our PAM approach and that longer WB are more often remembered (e.g. a longer walk in the park) than the exact number of shorter WBs.

Ultimately, the lack of correlation between the PAM macro-parameters and the clinical scores demonstrates that the everyday mobility of patients with APD and PD is a fully new perspective bringing a novel type of information compared to what can be observed and measured in the hospital with the clinical eye.

The added value of home monitoring compared to IGA or clinical scales is that it provides an objective measure of how the interplay between motor and non-motor impairments influence the daily activities of these patients. While micro parameters such as gait speed and stride length strongly correlate between IGA and PAM, particularly in long walking bouts, they provide no insight into the general activity of the patients, as no associations were found between these parameters. In fact, higher gait velocity does not necessarily indicate greater physical activity or a reduced fall risk. This is especially evident in patients with MSA, where results suggest more activity with lower MDS-UPDRS III scores, unlike in the other disease groups, independently of motor factors. For this reason, daily-life monitoring is paramount for fully characterizing and understanding patients’ conditions. PD, MSA, and PSP patients present very heterogeneous mobility profiles that do not always follow linear associations. Therefore, it is necessary to develop personalized digital mobility profiles and use them to monitor disease progression and evaluate response to therapy on an individual level.

Some limitations may potentially affect the results presented. First, the APD cohort shows a higher level of overall motor deficits, as expected and reflected by the H&Y staging. Since patients with APD manifest at an earlier age and exhibit significantly faster disease progression, matching for disease severity might result in significant discrepancies in age and disease duration in PD. Thus, accurate comparisons might not be possible due to the inhomogeneous groups which is to a certain extent considered as selection bias. However, this exploratory study provides a characterization of the three cohorts without adjusting for disease severity, which influences gait and balance. Consequently, the results should be interpreted primarily as insights into the gait and real-life mobility of these populations.

Second, the processing pipelines used to derive gait parameters from sensor signals were not validated specifically for MSA and PSP walking patterns due to the scarcity of these patients. Although these pipelines are based on kinematic principles—the validity of which is not linked to the subject’s clinical characteristics—a higher possibility of errors in the APD group remains.

Finally, during PAM, the participants were asked to wear the sensors upon awakening until bedtime. Therefore, the duration of the recordings might vary daily and between patients. However, no recording shorter than 8 h or longer than 12 h was included in the analysis. It is worth mentioning that adherence to the study protocol presented a greater challenge than dropping out due to adverse events. Ten patients with APD, corresponding to 18% of the population, were excluded due to insufficient amount of PAM recordings. This percentage is almost double that observed for PD (10.6%), highlighting important considerations for future studies aiming to conduct power analyses for real-life mobility monitoring in APD.

To conclude, this study comprehensively characterized the gait and mobility of a large, age- and sex-matched cohort of patients with MSA, PSP, and PD. The multi-perspective approach utilized in this study allows to gain a more complete understanding of how gait impairments, and clinical scores are associated with daily activity profiles in these populations. This knowledge is fundamental to improving and developing tailored management strategies towards precision medicine, which approaches patients´ homes instead of moving away from them and locating exclusively in hospitals. However, accurate reference values are required to provide healthcare professionals with the best means to interpret the data. This manuscript presents a set of evidence, but further research efforts are required in the future to expand these findings and understanding their clinical relevance (e.g. entropy) to ultimately translate them into innovative care procedures and outcome measures.

## Methods

Patients with probable MSA-P (here as MSA) according to second consensus criteria^31^, PSP-RS or PSP-P (here as PSP) according to the MDS-PSP criteria^32^, and patients with PD, according to MDS-PD diagnostic criteria^33^ were enrolled in this study, as part of the MobilityAPP trial (ClinicalTrials.gov, NCT04608604, date of registration: 19/10/2020), a therapeutic study assessing effectiveness of different physiotherapy interventions^34^. Patients were recruited between 01/02/2021 and 30/06/2024 from the inpatient and outpatient clinics of the neurology departments of the Medical University of Innsbruck, Austria; the University Hospital Erlangen, Germany; and the Regional Hospital of Bolzano, Italy. The study was approved by all local ethics committees (IRB numbers: 26_20 B (Erlangen), 1290/2020 (Innsbruck), 49-2015 (Bolzano), and all patients provided written informed consent prior to examination. To be eligible for the study, participants had to be between the ages of 30 and 80, on stable medication, able to walk independently (assistive devices such as a walker were allowed), and free of any major comorbidities that may influence the clinical presentation, mobility or gait, like recent fractures, orthopedic history or polyneuropathy. Patients with dementia, according to DSM-V, were excluded. A detailed list of inclusion and exclusion criteria is listed in the protocol manuscript^34^.

**Fig. 4:**
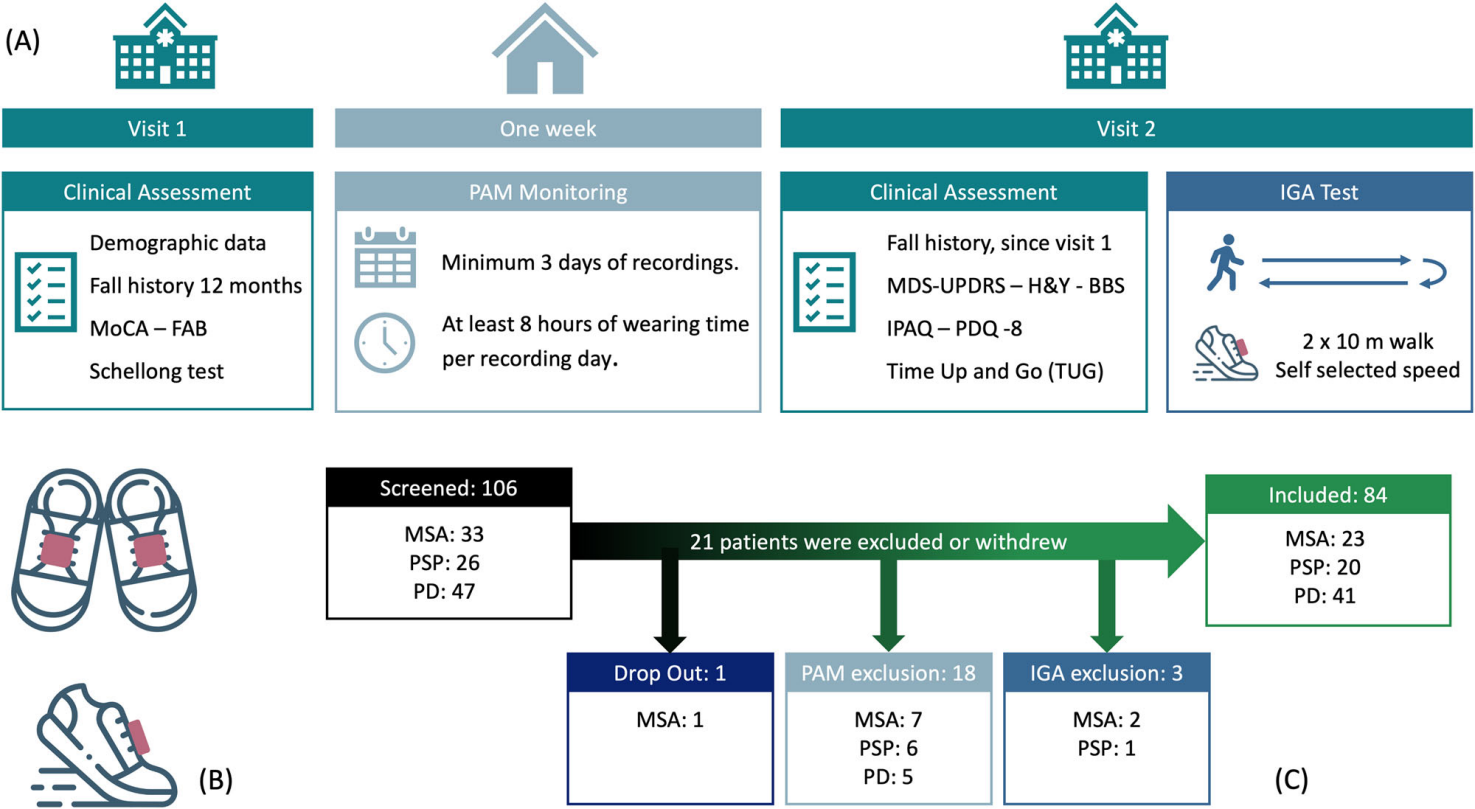
Data collection protocol and included participants. **A** Data collection protocol: patients visited the hospital twice (i.e., visit 1 and visit 2) with 1 week of PAM in between. **B** Sensor setup: IMUs were positioned on the instep of the feet during IGA and PAM. **C** Study Flowchart of included patients.

All patients underwent two visits, with 1 week of physical activity monitoring (PAM) at home in between. While visit 1 recorded demographics, fall history, and non-motor features such as orthostatic hypotension (OH) and cognition, visit 2 was used for motor assessments, questionnaires, and an instrumented gait analysis (IGA) with a standardized two-times-ten-meters (2x10m) gait test at self-selected speed. During the visits, rating scales and IGA were performed in ON state (see Fig. 4).

### Clinical data

At visit 1, the basic demographic data, including age, sex, height, weight, disease duration, and concomitant medication, including the levodopa-equivalent daily dose (LEDD), were obtained. Patients were asked about previous falls and grouped as non-fallers (maximum of one fall within the last 12 months) and fallers (more than one fall)^35^. All patients were clinically staged by modified Hoehn & Yahr score (H&Y). Cognition was tested using the Montreal Cognitive Assessment (MoCA)^36^ and the Frontal Assessment Battery (FAB)^37^. Additionally, all participants performed an orthostatic standing test, where a minimum drop of 20 mmHg in systolic blood pressure or 10 mmHg in diastolic blood pressure within 3 min of standing was defined as OH^38^.

After 1 week of PAM, patients returned for the second visit, where the Unified Parkinson’s Disease Rating Scale (MDS-UPRDS)^39^ was administered. The postural instability and gait difficulty (PIGD) score was derived from the sum of the gait, postural stability arising from the chair, and posture items measured in MDS-UPDRS-III^15^. Balance control was assessed using the Berg Balance Scale (BBS)^40^, and the timed up and go (TUG) test was performed to measure physical mobility^41,42^. Finally, PROMs were complemented by questionnaires about quality of life (PDQ-8)^43^ and physical activity (IPAQ)^44^.

### IGA in the clinic

During visit 2, patients performed an IGA wearing the IMU-based mobile GaitLab system (Portabiles HealthCare Technologies GmbH, Erlangen, Germany) on the instep of their shoes (Fig. 1B). Each IMU is equipped with a 3D accelerometer and a 3D gyroscope with a sampling rate of 102.4 Hz. Participants were instructed to perform a 2x10m test. Using a pipeline based on the Gaitmap package^45^, five spatiotemporal gait features were calculated for each detected stride: gait velocity, stride length, stride time, stance percentage, and maximum sensor lift. Stance percentage was calculated as the ratio of stance time to stride time, and maximum sensor lift was defined as the maximum height that the sensor reaches from the position where the foot lies flat on the ground.

The mean and coefficient of variation (CV) for each parameter were obtained across all strides on both feet. The mean provides information about the average gait pattern, while the CV measures inter-stride variability during the test. Moreover, an asymmetry index was calculated for all pairs of consecutive left and right strides according to Eq. (1)^46^:

1 $$Asymmetry=Mdn\left(\left|\frac{{P}_{Li}-{P}_{Ri}\,}{{P}_{Li}+{P}_{Ri}\,}\right|\times 100\right)\mathrm{for}\,i\in \left[1,Num\,Steps\right]$$

In Eq. (1), ${P}_{{Li}}$ and ${P}_{{Ri}}$ represent the gait feature values at the step “*i*” for the left and right foot, respectively, while the Mdn is the median of the sequence. A larger asymmetry value indicates a greater difference between the sides.

### PAM at home

The participants’ daily-life mobility was recorded between visits 1 and 2 using the same wearable devices as for the IGA. Patients were trained to use the sensor system and instructed to wear them for 7 consecutive days, from awakening until bedtime. Following pre-defined criteria^47^, patients were considered in the PAM analysis only if a minimum of 3 days with 8 h of wearing time per day was available. The non-wearing period detection was based on a triple threshold on accelerometer norm (less than 0.05 g), gyroscope norm (less than 2 degree/s), and time window (more than 90 min of inactivity).

The processing of PAM data was based on walking bouts (WBs), i.e., a sequence of consecutive steps. Three types of WB were defined according to the walking duration: short WB between 10 and 30 seconds, medium WB between 30 and 60 s, and long WB longer than 60 s^48^. Parameters from two different types of metrics were extracted using a previously validated pipeline^49^: physical activity (PA) metrics, defined as “macro-parameters,” and gait metrics, described as “micro-parameters” (see Supplementary Table 1 for additional information)^35,50^.

Two different aggregation methods were used for macro and micro parameters (see Supplementary Fig. 1). PA parameters were computed per day of recording, and ultimately, the median and maximum values across the recording days were used respectively as descriptors of the participant’s average and maximum PA performance. The primary outcomes include the median number of walking bouts, categorized as short, medium, and long; the duration and maximum number of long WBs; step count; the ratio of active to sedentary percentage; and the daily moderate-to-vigorous physical activity (MVPA). A threshold of 90 steps per minute was chosen as an appropriate cut-off between light PA and MVPA^51^.

Micro parameters were assessed throughout the entire recording period. Three gait metrics were considered: velocity, stride length, and cadence. Their distributions were used to model probability density functions (PDFs) through a non-parametric kernel density smoothing algorithm. Finally, the mode of the PDFs (i.e., the most probable value to occur) was considered the patient’s usual gait pattern, while the 95th percentile represented the maximum performance the participant achieved in daily-life. To ensure a proper estimation of the PDFs, micro parameters were computed only if the number of WBs available was larger than 10.

In addition to the macro and micro parameters, the complexity metric called *“information entropy”* (Hn)^35^ was computed. This measurement quantifies the variety of performed tasks by the participants during their daily lives, classifying mobility profiles into 25 different states according to the type of activity (locomotion, non-locomotion), duration (very short, short, medium, long), and intensity (acceleration magnitude, cadence)^52^. Larger values of Hn are associated with a greater variety of PA states and an enhanced ability to transition between them, ultimately reflecting a higher mobility in the patient.

### Statistical analysis

Statistical tests were performed among the three groups of patients regarding clinical measurements, IGA, and PAM. The normality of residuals within each distribution was tested with the Shapiro-Wilk test, as well as the homogeneity of variances among the groups was assessed with Levene’s test. One-way ANOVA was applied to parameters with normally distributed residuals and homogenous variances. In cases where residuals were non-normally distributed or the variances were heterogeneous, the Kruskal–Wallis test was used. To control the false discovery rate (FDR) associated with multiple testing, the calculated *p*-values were adjusted using the Benjamini-Hochberg correction. The FDR threshold (*α*) was set at 0.05, and eta squared (*η*²) was used to report the effect size. Post hoc analyses were performed for tests with *p* values below the threshold (*α* < 0.05). Independent *t*-tests were conducted for parameters analyzed with ANOVA; otherwise, Dunn’s test was employed. The significance level (*α*) for post hoc analysis was also set at 0.05.

Spearman coefficients (*ρ*) were computed for each patient group to evaluate the correlation between clinical scores and sensor-derived parameters. An associated *p* value lower than 0.05 determined the significance of the correlation. Correlations with *ρ* ≤ 0.25 were classified as small, 0.25–0.50 as moderate, 0.50 − 0.75 as good, and ≥0.75 as excellent. The statistical analysis was performed using R version 4.3.1 on the “aarch64-apple-darwin20” platform.

### Ethical standard

All procedures performed in studies involving human participants were in accordance with the ethical standards of the institutional and/or national research committee and with the 1975 Helsinki declaration and its later amendments or comparable ethical standards. Informed consent was obtained from all the patients included in the study.

## Supplementary information

SupplementaryInformation.

## Data Availability

The data sets analyzed during this study are available from the corresponding authors upon reasonable request.

## Code availability

The underlying code for this study is not publicly available but may be made available to qualified researchers on reasonable request from the corresponding author.
