# Supplementary material for: Moving beyond the hospital: in-depth characterization of daily-life mobility in patients with atypical Parkinsonian disorders
Source: NPJ Parkinsons Dis. 2026 Jan 12;12:34. doi: 10.1038/s41531-025-01242-2 (PMC12858815; doi:10.1038/s41531-025-01242-2)
Supplement: Supplementary file 1 — SupplementaryInformation. [file 41531_2025_1242_MOESM1_ESM.pdf]

# Supplementary Material

## Supplementary Tables

Supplementary Table 1: description of PAM micro and macro parameters

| Feature | Type | Unit of Measurement | Definition |
|---|---|---|---|
| Mode Gait Velocity Short WB | Micro | m/s | Mode of the weekly distribution of the gait velocity feature from WBs between 10s and 30s of duration |
| Mode Gait Velocity Medium WB | Micro | m/s | Mode of the weekly distribution of the gait velocity feature from WBs between 30s and 60s of duration |
| Mode Gait Velocity Long WB | Micro | m/s | Mode of the weekly distribution of the gait velocity feature from WBs above 60s of duration |
| 95th Percentile Gait Velocity All WB | Micro | m/s | 95th of the weekly distribution of the gait velocity feature from all WBs |
| Mode Stride Length Short WB | Micro | m | Mode of the weekly distribution of the stride length feature from WBs between 10s and 30s of duration |
| Mode Stride Length Medium WB | Micro | m | Mode of the weekly distribution of the stride length feature from WBs between 30s and 60s of duration |
| Mode Stride Length Long WB | Micro | m | Mode of the weekly distribution of the stride length feature from WBs above 60s of duration |
| 95th Percentile Stride Length All WB | Micro | m | 95th of the weekly distribution of the stride length feature from all WBs |
| Mode Cadence Short WB | Micro | steps/min | Mode of the weekly distribution of the cadence feature from WBs between 10s and 30s of duration |
| Mode Cadence Medium WB | Micro | steps/min | Mode of the weekly distribution of the cadence feature from WBs between 30s and 60s of duration |
| Mode Cadence Long WB | Micro | steps/min | Mode of the weekly distribution of the cadence feature from WBs above 60s of duration |
| Median Number of Short WB / day | Macro | WB/day | Weekly median of daily sum of number of WBs between 10s and 30s of duration |
| Median Number of Medium WB / day | Macro | WB/day | Weekly median of daily sum of number of WBs between 30s and 60s of duration |
| Median Number of Long WB / day | Macro | WB/day | Weekly median of daily sum of number of WBs longer than 60s of duration |
| Max Number of Long WB / day | Macro | WB/day | Weekly maximum of daily sum of number of WBs longer than 60s of duration |
| Median Duration of Long WB / day | Macro | s/day | Weekly median of daily average duration of WBs longer than 60s of duration |
| Median Step count/day | Macro | step/day | Weekly median of daily sum of steps using all WB |
| Median Ratio Active/Sedentary | Macro | - | Weekly median of the ratio between active and sedentary time measured |
| Median MVPA Time | Macro | min/day | Weekly median of the daily time spent in moderate-to-vigorous activity |
| Median Hn | Complexity | Hn/day | Weekly median of daily entropy measured in activity patterns |

Supplementary table 2: correlation analysis between the 2x10m test and clinical scores

| Parameter | MDS-UPDRS III | | | PIGD | | | BBS | | |
|---|---|---|---|---|---|---|---|---|---|
| | **ρ MSA** | **ρ PSP** | **ρ PD** | **ρ MSA** | **ρ PSP** | **ρ PD** | **ρ MSA** | **ρ PSP** | **ρ PD** |
| **Mean** | | | | | | | | | |
| Gait Velocity (m/s) | -0.40 | **-0.67*** | **-0.48*** | **-0.62*** | **-0.71*** | **-0.50*** | **0.81*** | **0.47*** | 0.27 |
| Stride Length (m) | **-0.44*** | **-0.61*** | **-0.44*** | **-0.61*** | **-0.65*** | **-0.49*** | **0.80*** | **0.57*** | **0.39*** |
| Stride Time (s) | 0.05 | 0.30 | 0.31 | 0.27 | 0.34 | **0.31*** | -0.35 | -0.21 | 0.00 |
| Stance Percentage | 0.18 | **0.50*** | 0.06 | 0.41 | **0.55*** | 0.19 | **-0.69*** | -0.33 | 0.03 |
| Max Sensor Lift (m) | -0.30 | -0.20 | 0.06 | -0.28 | -0.23 | 0.19 | **0.69*** | 0.04 | 0.21 |
| **Coefficient of Variation** | | | | | | | | | |
| Gait Velocity | 0.15 | **0.54*** | **0.41*** | 0.18 | **0.51*** | **0.57*** | **-0.67*** | -0.45 | **-0.49*** |
| Stride Length | 0.14 | 0.37 | **0.34*** | 0.17 | 0.33 | **0.52*** | **-0.64*** | -0.42 | **-0.43*** |
| Stride Time | -0.09 | **-0.52*** | **0.51*** | 0.09 | **0.59*** | **0.60*** | **-0.51*** | -0.37 | **-0.56*** |
| Stance Percentage | 0.03 | 0.41 | 0.28 | 0.14 | **0.62*** | **0.38*** | **-0.7*** | **-0.61*** | **-0.35*** |
| Max Sensor Lift | -0.16 | 0.39 | -029 | -0.08 | **0.57*** | -0.12 | -0.43 | -0.36 | 0 |
| **Asymmetry (%)** | | | | | | | | | |
| Gait Velocity | 0.05 | **0.50*** | 0.12 | 0.02 | **0.47*** | 0.19 | -0.3 | -0.24 | **-0.31*** |
| Stride Length | 0.01 | **0.53*** | 0.08 | -0.12 | **0.46*** | 0.13 | **-0.44*** | -0.28 | 0.21 |
| Stride Time | 0.16 | **0.46*** | -0.13 | **0.43*** | **0.57*** | 0.07 | -0.37 | -0.27 | -0.26 |
| Stance Percentage | 0.27 | 0.04 | -0.13 | 0.38 | 0.28 | 0.23 | **-0.52*** | -0.38 | 0.1 |
| Max Sensor Lift | -0.32 | 0.28 | **-0.32*** | -0.26 | 0.44 | -0.23 | -25 | -0.34 | 0.11 |

ρ values represent Spearman correlation coefficients. Bold numbers with * indicate significant correlations ($p<0.05$). MDS-UPDRS III: Movement disorder society Unified Parkinson's disease rating scale-part 3; PIGD: Postural instability and gait difficulty; BBS: Berg balance scale.

Supplementary table 3: correlation analysis between the physical activity monitoring and clinical scores

| Parameter | MDS-UPDRS III | | | PIGD | | | IPAQ Walking | | |
|---|---|---|---|---|---|---|---|---|---|
| | ρ MSA | ρ PSP | ρ PD | ρ MSA | ρ PSP | ρ PD | ρ MSA | ρ PSP | ρ PD |
| **Short WBs (10-30 s)** | | | | | | | | | |
| Gait Velocity | **-0.60*** | -0.36 | -0.12 | -0.4 | -0.29 | **-0.47*** | -0.33 | **0.48*** | 0.13 |
| Stride Length | **-0.62*** | -0.08 | 0.00 | **-0.56*** | 0.01 | **-0.34*** | -0.16 | **0.52*** | 0.17 |
| Cadence | 0.08 | -0.18 | -0.19 | 0.12 | -0.23 | -0.21 | **-0.46*** | -0.34 | 0.05 |
| No. of WB/day | -0.27 | -0.42 | -0.23 | -0.06 | -0.39 | -0.13 | **-0.47*** | -0.19 | 0.03 |
| **Medium WBs (31-60 s)** | | | | | | | | | |
| Gait Velocity | **-0.64*** | -0.19 | -0.18 | **-0.53*** | -0.02 | **-0.43*** | -0.28 | 0.10 | 0.30 |
| Stride Length | **-0.56*** | -0.09 | 0.01 | **-0.52*** | -0.09 | -0.25 | -0.01 | 0.30 | 0.26 |
| Cadence | -0.23 | -0.07 | -0.19 | -0.06 | 0.03 | -0.30 | -0.39 | -0.39 | 0.15 |
| No. of WB/day | **-0.49*** | -0.11 | -0.34 | -0.26 | -0.22 | -0.15 | -0.36 | -0.22 | -0.04 |
| **Long WBs (> 60 s)** | | | | | | | | | |
| Gait Velocity | **-0.48*** | **-0.63*** | -0.21 | -0.37 | **-0.54*** | **-0.42*** | -0.25 | 0.28 | **0.35*** |
| Stride Length | **-0.58*** | **-0.56*** | -0.12 | -0.43 | **-0.49*** | **-0.37*** | -0.01 | **0.49*** | 0.23 |
| Cadence | -0.33 | -0.43 | -0.23 | -0.22 | -0.38 | -0.29 | -0.12 | -0.01 | 0.19 |
| No. of WB/day | -0.32 | 0.01 | 0.05 | -0.23 | 0.22 | 0.08 | 0.41 | **0.51*** | **0.31*** |
| Max. No. of WB/day | -0.1 | -0.14 | 0.24 | 0.10 | -0.13 | 0.18 | **0.48*** | **0.49*** | **0.32*** |
| Median duration | -0.41 | -0.4 | 0.18 | -0.39 | -0.22 | 0.13 | 0.32 | **0.47*** | **0.36*** |
| **95th percentile all WBs** | | | | | | | | | |
| Gait Velocity | **-0.65*** | **-0.73*** | -0.26 | **-0.62*** | **-0.50*** | **-0.37*** | 0.28 | 0.33 | 0.08 |
| Stride Length | **-0.65*** | **-0.68*** | -0.22 | **-0.71*** | -0.44 | **-0.36*** | 0.39 | 0.38 | 0.12 |
| **Macro and complexity parameters (median)** | | | | | | | | | |
| Step count / day | -0.40 | -0.31 | -0.21 | -0.22 | -0.33 | -0.11 | 0.03 | -0.03 | 0.26 |
| Ratio Active/Sedentary | **-0.56*** | -0.39 | -0.13 | -0.37 | -0.42 | -0.10 | -0.09 | -0.01 | 0.20 |
| MVPA | -0.39 | -0.37 | -0.24 | -0.17 | -0.40 | -0.15 | -0.01 | -0.08 | 0.22 |
| Information Entropy | **-0.58*** | **-0.55*** | -0.21 | -0.36 | **-0.52*** | -0.20 | -0.01 | -0.02 | 0.23 |

ρ ρ values represent Spearman correlation coefficients. Bold numbers with * indicate significant correlations (p<0.05). MDS-UPDRS III: Movement disorder society Unified Parkinson’s disease rating scale-part 3; PIGD: Postural instability and gait difficulty; IPAQ: International Physical Activity Questionnaire.

# Supplementary Figures

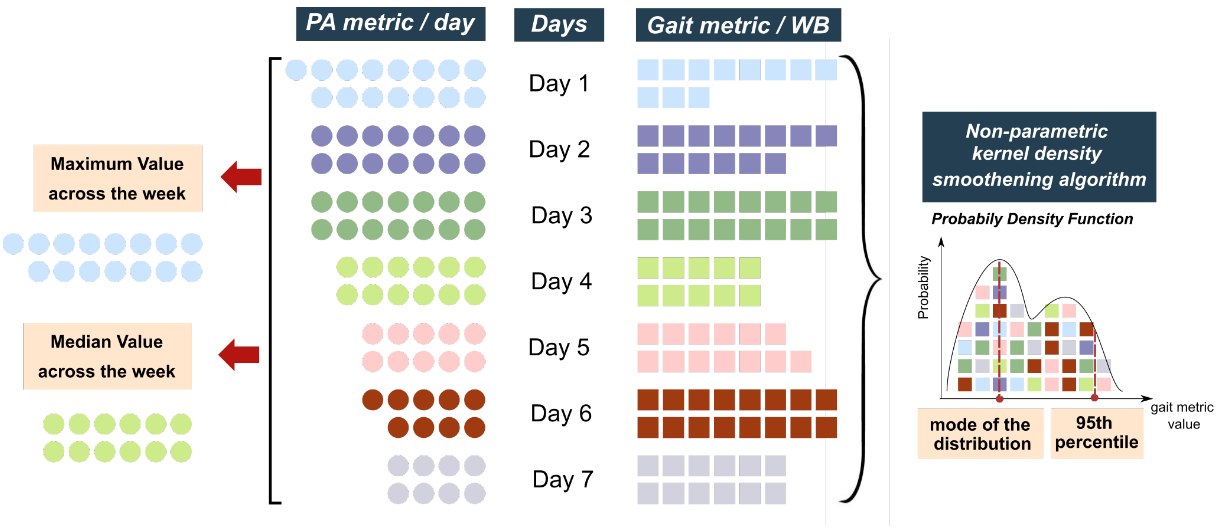


Supplementary Figure 1: aggregation method for PAM micro and macro parameters.

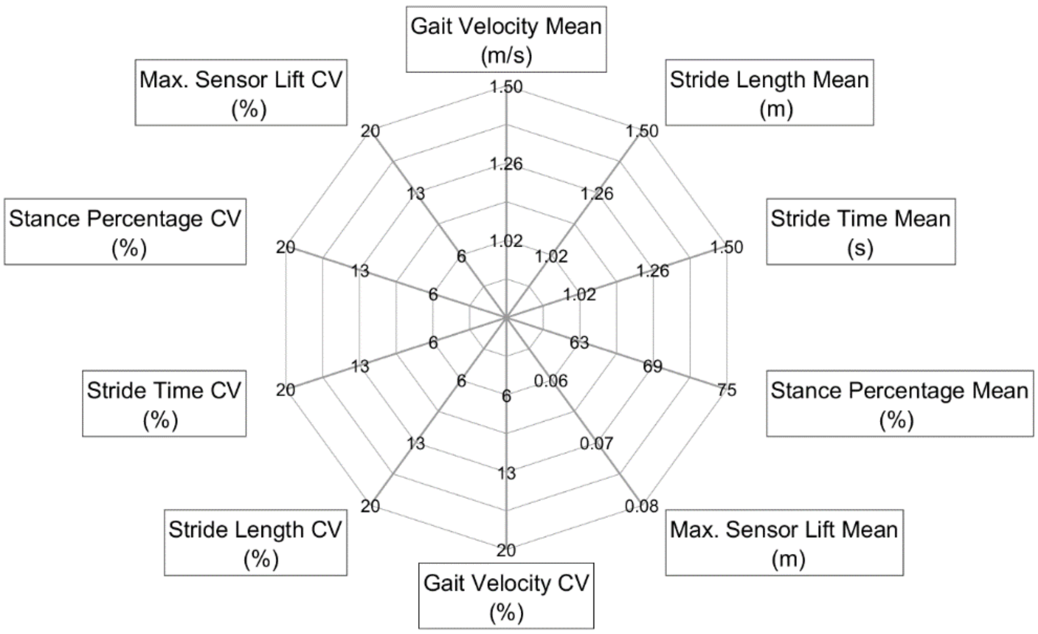


Supplementary Figure 2: Unit of measurement of IGA spider plot (Fig. 1).

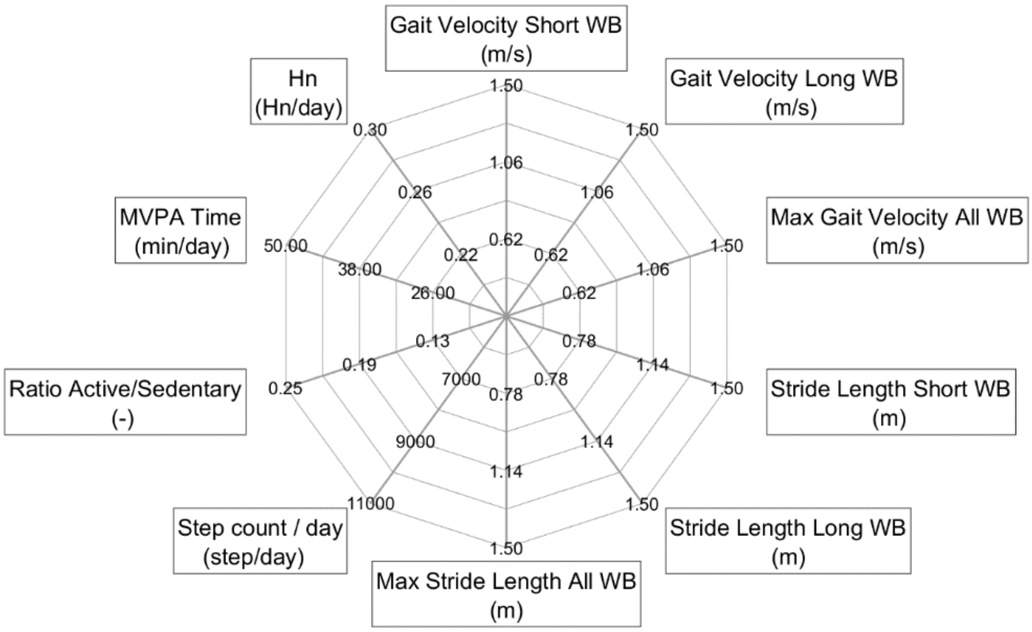


Supplementary Figure 3: Unit of measurement of PAM spider plot (Fig. 2).

| Name | Location | Affiliation | Role | Contribution | Mail | Author (A) or Coinvestigator (C) |
|---|---|---|---|---|---|---|
| Patrick Bachmann | Medical University of Innsbruck | Department of Neurology, Medical University of Innsbruck, Austria | Site Investigator | Visit scheduling and data management at MUI | patrick.bachmann@i-med.ac.at | C |
| Georg Göbel | Medical University of Innsbruck | Department of Neurology, Medical University of Innsbruck, Austria | Statistician | Providing statistical plan and analysis | georg.goebel@i-med.ac.at | A |
| Helene Humer | Medical University of Innsbruck | Department of Neurology, Medical University of Innsbruck, Austria | Site Investigator | Visit scheduling and data management at MUI | helene.humer@i-med.ac.at | C |
| Frank Jagusch | Medical University of Innsbruck | Department of Neurology, Medical University of Innsbruck, Austria | Site Investigator | conducting recruitment and study visits | frank.jagusch@tirol-kliniken.at | A |
| Florian Krismer | Medical University of Innsbruck | Department of Neurology, Medical University of Innsbruck, Austria | PI | PI of the study | florian.krismer@i-med.ac.at | A |
| Jean-Pierre Ndayisaba | Medical University of Innsbruck | Department of Neurology, Medical University of Innsbruck, Austria | Study coordinator | data management, statistical analysis | jean-pierre.ndayisaba@i-med.ac.at | A |
| Gudrun Schoenherr | Medical University of Innsbruck | Department of Neurology, Medical University of Innsbruck, Austria | Site Investigator | Responsible for coordinating physiotherapists | gudrun.schoenherr@tirol-kliniken.at | C |
| Stefan Kiechl | Medical University of Innsbruck | Department of Neurology, Medical University of Innsbruck, Austria | Site Investigator | Former Chief of Neurology Department, Coordination | stefan.kiechl@tirol-kliniken.at | C |
| Werner Poewe | Medical University of Innsbruck | Department of Neurology, Medical University of Innsbruck, Austria | Site Investigator | Chief of Neurology Department, Coordination | werner.poewe@i-med.ac.at | C |
| Johanna Wüstner | Medical University of Innsbruck | Department of Neurology, Medical University of Innsbruck, Austria | Physiotherapist | Physiotherapist | johenna.wüstner@tirol-kliniken.at | C |
| Anna Resch | Medical University of Innsbruck | Department of Neurology, Medical University of Innsbruck, Austria | Physiotherapist | Physiotherapist | anna.resch@tirol-kliniken.at | C |
| Svenja Schmidt | Medical University of Innsbruck | Department of Neurology, Medical University of Innsbruck, Austria | Physiotherapist | Physiotherapist | svenja.schmidt@tirol-kliniken.at | C |
| Pascalle Hendriks | Medical University of Innsbruck | Department of Neurology, Medical University of Innsbruck, Austria | Physiotherapist | Physiotherapist | pascalle.hendriks@tirol-kliniken.at | C |
| Raphaela Greimann | Medical University of Innsbruck | Department of Neurology, Medical University of Innsbruck, Austria | Physiotherapist | Physiotherapist | raphaela.greimann@tirol-kliniken.at | C |
| Hannah Huber | Medical University of Innsbruck | Department of Neurology, Medical University of Innsbruck, Austria | Physiotherapist | Physiotherapist | hannah.huber@tirol-kliniken.at | C |
| Klaus Seppi | Medical University of Innsbruck, Regional Hospital Kufstein, Austria | Department of Neurology, Medical University of Innsbruck, Austria Regional Hospital Kufstein, Austria | Site Investigator | project planning, patients recruitment | klaus.seppi@i-med.ac.at | A |
| Victoria Sidoroff | Medical University of Innsbruck | Department of Neurology, Medical University of Innsbruck, Austria | Site Investigator | coordination, project planning, patients recruitment | victoria.sidoroff@tirol-kliniken.at | A |
| Gregor Wenning | Medical University of Innsbruck | Department of Neurology, Medical University of Innsbruck, Austria | PI | former PI of the study | xxx | A |
| Heiko Gaßner | Uniklinikum Erlangen | Department of Molecular Neurology, Uniklinikum Erlangen | PI | project planning and monitoring, study coordination Erlangen, statistical analysis | heiko.gassner@uk-erlangen.de | A |
| Jürgen Winkler | Uniklinikum Erlangen | Department of Molecular Neurology, Uniklinikum Erlangen | Co-PI | project planning and monitoring, patient recruitment | juergen.winkler@uk-erlangen.de | A |
| Isabelle Teckenburg | Uniklinikum Erlangen | Department of Molecular Neurology, Uniklinikum Erlangen | Site Investigator | patient recruitment, exercise intervention and study assessments, data management | isabelle.teckenburg@uk-erlangen.de | A |
| Kathrin Kinscher | Uniklinikum Erlangen | Department of Molecular Neurology, Uniklinikum Erlangen | Site Investigator | patient recruitment, exercise intervention and study assessments | kathrin.kinscher@uk-erlangen.de | C |
| Nina Hergenröder-Lenzner | Uniklinikum Erlangen | Department of Molecular Neurology, Uniklinikum Erlangen | Site Investigator | patient recruitment, exercise intervention and study assessments | nina.hergenroeder-lenzner@uk-erlangen. | A |
| Martin Regensburger | Uniklinikum Erlangen | Department of Molecular Neurology, Uniklinikum Erlangen | Site Investigator | patient recruitment, clinical study assessments | martin.regensburger@uk-erlangen.de | C |
| Jelena Jukic | Uniklinikum Erlangen | Department of Molecular Neurology, Uniklinikum Erlangen | Site Investigator | patient recruitment, clinical study assessments | jelena.jukic@uk-erlangen.de | C |
| Sabine Stallforth | Uniklinikum Erlangen | Department of Molecular Neurology, Uniklinikum Erlangen | Site Investigator | patient recruitment, clinical study assessments | sabine.stallforth@uk-erlangen.de | C |
| Alexander German | Uniklinikum Erlangen | Department of Molecular Neurology, Uniklinikum Erlangen | Site Investigator | patient recruitment, clinical study assessments | alexander.german@uk-erlangen.de | C |
| Christina Erhardt | Uniklinikum Erlangen | Department of Molecular Neurology, Uniklinikum Erlangen | Site Investigator | patient recruitment, clinical study assessments | christina.erhardt@uk-erlangen.de | C |
| Patrick Süß | Uniklinikum Erlangen | Department of Molecular Neurology, Uniklinikum Erlangen | Site Investigator | patient recruitment, clinical study assessments | patrick.suess@uk-erlangen.de | C |
| Emily Adler | Uniklinikum Erlangen | Department of Molecular Neurology, Uniklinikum Erlangen | Site Investigator | study assessments, data management | emily.adler@uk-erlangen.de | C |
| Andrea Weitzenfelder | Uniklinikum Erlangen | Department of Molecular Neurology, Uniklinikum Erlangen | Administrative Support | Visit scheduling, support study coordination | andrea.weitzenfelder@uk-erlangen.de | C |
| Cecilia Raccagni | Hospital of Bolzano (SABES-ASDAA) | Hospital of Bolzano (SABES-ASDAA), Department of Neurology, Bolzano-Bozen, Italy, Teaching Hospital of Paracelsus Medical University | PI | Study author and coordinator, patients recruitment, data management at Bolzano | cecilia.raccagni@sabes.it | A |
| Ilaria Scarano | Hospital of Bolzano (SABES-ASDAA) | Hospital of Bolzano (SABES-ASDAA), Department of Rehabilitation, Merano-Meran, Italy, Teaching Hospital of Paracelsus Medical University | Site Investigator | patients recruitment, scheduling visits at Bolzano | giudittailaria.scarano@sabes.it | C |
| Michela Peranzoni | Hospital of Bolzano (SABES-ASDAA) | Hospital of Bolzano (SABES-ASDAA), Department of Neurology, Bolzano-Bozen, Italy, Teaching Hospital of Paracelsus Medical University | Physiotherapist | Coordination of physiotherapists of Bolzsno | michela.peranzoni@sabes.it | C |
| Barbara Zaniboni | Hospital of Bolzano (SABES-ASDAA) | Hospital of Bolzano (SABES-ASDAA), Department of Neurology, Bolzano-Bozen, Italy, Teaching Hospital of Paracelsus Medical University | Physiotherapist | Physiotherapist | barbara.zaniboni@sabes.it | C |
| Karin Voelkl | Hospital of Bolzano (SABES-ASDAA) | Hospital of Bolzano (SABES-ASDAA), Department of Neurology, Bolzano-Bozen, Italy, Teaching Hospital of Paracelsus Medical University | Physiotherapist | Physiotherapist | karin.voelkl@sabes.it | C |
| Martina Bortolini | Hospital of Bolzano (SABES-ASDAA) | Hospital of Bolzano (SABES-ASDAA), Department of Neurology, Bolzano-Bozen, Italy, Teaching Hospital of Paracelsus Medical University | Site Investigator | patients recruitment, scheduling visits at Bolzano | martina.bortolini97@gmail.com | C |
| Anisoara Paraschiv-Ionescu | Ecole Polytechnique Federale de Lausanne | Laboratory of Movement Analysis and Measurement, Ecole Polytechnique Federale de Lausanne, Switzerland | Site Investigator | Analysis of PAM data, Data Analysis planning, | anisoara.ionescu@epfl.ch | A |
| Gaelle Prigent | Ecole Polytechnique Federale de Lausanne | Laboratory of Movement Analysis and Measurement, Ecole Polytechnique Federale de Lausanne, Switzerland | Site Investigator | Analysis of PAM data, Data Analysis planning, | gaelle.prigent10@gmail.com | A |
| Kamiar Aminian | Ecole Polytechnique Federale de Lausanne | Laboratory of Movement Analysis and Measurement, Ecole Polytechnique Federale de Lausanne, Switzerland | PI | PI of the study | kamiar.aminiangepfl.ch | A |
| Bjoern Eskofier | Erlangen | Machine Learning and Data Analytics Lab, Friedrich-Alexander-Universität Erlangen-Nürnberg, Germany / Translational Digital Health Group, Institute of AI for Health, Helmholtz Zentrum München - German Research Center for Environmental Health, Neuherberg, Germany | PI | PI of the study | bjoern.eskofier@fau.de | A |
| Hamid Moradi | Erlangen | Machine Learning and Data Analytics Lab, Friedrich-Alexander-Universität Erlangen-Nürnberg, Germany | PhD student and Data Scientist | algorithm implementation and data analysis | hamid.moradi@fau.de | A |
| Marzieh Asalian | Erlangen | Machine Learning and Data Analytics Lab, Friedrich-Alexander-Universität Erlangen-Nürnberg, Germany | Master student and Data Scientist (contributing role) | Data management and quality check | marzieh.asalian@fau.de | A |
| Nils Roth | Erlangen | Machine Learning and Data Analytics Lab, Friedrich-Alexander-Universität Erlangen-Nürnberg, Germany | PhD student and Data Scientist (graduated) | algorithm implementation and data analysis | nils.roth@fau.de | C |
| Lisann Lieb | Erlangen | Machine Learning and Data Analytics Lab, Friedrich-Alexander-Universität Erlangen-Nürnberg, Germany | Data Scientist (contributing role) | data analysis support | lisann.lieb@fau.de | C |
| Felix Kluge | Erlangen | Machine Learning and Data Analytics Lab, Friedrich-Alexander-Universität Erlangen-Nürnberg, Germany | PostDoc and Resaerch Group Leader | data analysis coordination | felix.kluge@fau.de | C |
| Jochen Klucken | University of Luxembourg | Luxembourg Centre for Systems Biomedicine, University of Luxembourg, Esch-sur-Alzette, Luxembourg<br>Luxembourg Institute of Health, Strassen, Luxembourg<br>Centre Hospitalier de Luxembourg, rollengergronn-belair-nord, Luxembourg | PI | PI of the Luxembourg site | jochen.klucken@uni.lu | A |
| Marijus Giraitis | University of Luxembourg | Luxembourg Centre for Systems Biomedicine, University of Luxembourg, Esch-sur-Alzette, Luxembourg<br>Luxembourg Institute of Health, Strassen, Luxembourg<br>Centre Hospitalier de Luxembourg, rollengergronn-belair-nord, Luxembourg | Site Investigator | conducting recruitment and study visits | marijus.giraitis@uni.lu | A |
| Gelani Zelimkhanov | University of Luxembourg | Luxembourg Centre for Systems Biomedicine, University of Luxembourg, Esch-sur-Alzette, Luxembourg<br>Luxembourg Institute of Health, Strassen, Luxembourg<br>Centre Hospitalier de Luxembourg, rollengergronn-belair-nord, Luxembourg | Site Investigator | conducting recruitment and study visits | gelani.zelimkhanov@ext.uni.lu | C |
| Olena Tsurkalenko | University of Luxembourg | Luxembourg Centre for Systems Biomedicine, University of Luxembourg, Esch-sur-Alzette, Luxembourg<br>Luxembourg Institute of Health, Strassen, Luxembourg<br>Centre Hospitalier de Luxembourg, rollengergronn-belair-nord, Luxembourg | Site Investigator | conducting recruitment and study visits | olena.tsurkalenko@lih.lu | C |
| Stefano Sapienza | University of Luxembourg | Luxembourg Centre for Systems Biomedicine, University of Luxembourg, Esch-sur-Alzette, Luxembourg<br>Luxembourg Institute of Health, Strassen, Luxembourg | Data scientist | Analysing data | stefano.sapienza@uni.lu | A |
| David Benninger | Switzerland | Neurology, Centre Hospitalier Universitaire Vaudois, Lausanne, Switzerland<br>Université de Lausanne, Lausanne, Switzerland<br>Neurology, Reha Rheinfelden, Rheinfelden, Switzerland | PI | PI of CHUV/Reha Rheinfelden | david.benninger@chuv.ch | A |
| -Tabea Schoenfeldt-Reichm | CHUV | Neurology, Centre Hospitalier Universitaire Vaudois, Lausanne, Switzerland<br>Université de Lausanne, Lausanne, Switzerland | Site investigator | oordination, conducting recruitment and study visit | va-Tabea.Schoenfeldt-Reichmann@chuv.c | A |
